# Tricuspid Regurgitation: A Comprehensive Review of Clinical, Imaging and Therapy

**DOI:** 10.31083/RCM28173

**Published:** 2025-05-08

**Authors:** Frank F. Seghatol-Eslami, Kan Liu

**Affiliations:** ^1^Division of Cardiology, Department of Medicine, Washington University School of Medicine, St. Louis, MO 63110-1083, USA

**Keywords:** tricuspid valve, right ventricle, transesophageal and interventional echocardiography, medical therapy, surgical intervention, transcatheter edge-to-edge repair and valve replacement

## Abstract

Once considered the “forgotten valve and ventricle”, the tricuspid valve and right ventricle are now recognized as critical structures with significant clinical and prognostic implications. Growing evidence has highlighted that tricuspid regurgitation (TR) and right heart failure are not merely secondary phenomena that resolve following the treatment of left-sided heart disease. Instead, TR and right heart failure contribute to adverse outcomes and increased mortality if left untreated. This paradigm shift has fueled extensive clinical research, leading to a deeper understanding of the pathophysiology of TR and right ventricular (RV) dysfunction. Additionally, advancements in cardiovascular imaging have facilitated early detection, risk stratification, and innovative therapeutic approaches for TR and right heart failure. This article explores the evolving landscape of tricuspid valve disease, emphasizing the importance of early recognition and the role of emerging imaging technologies in improving patient outcomes. Thanks to progress in imaging technology, especially echocardiography, as well as cardiac magnetic resonance and cardiac computer tomography, enhanced studies can be conducted on the tricuspid valve pathology to delineate the various mechanisms involved in TR and RV dysfunction and offer patients a tailored medical, as well as surgical and transcatheter therapies. These unparalleled technological advances would not be possible without the hard work of physicians, scientists, surgeons, interventional cardiologists, and echocardiographers worldwide, despite the many challenges they experience daily and in every procedure. Many patients with TR present at an advanced stage of disease progression, often with severe regurgitation and clinical manifestations associated with poor outcomes. Additionally, a significant proportion of these patients have either undergone previous open-heart surgery for left-sided valvular disease or are considered high-risk surgical candidates due to multiple comorbid conditions. In recent years, transcatheter therapy has emerged as a viable alternative for this high-risk population, offering a less invasive option for those previously deemed “inoperable”. This breakthrough has transformed the therapeutic landscape for valvular heart disease, particularly for TR, providing new hope and improved outcomes for patients who were once left with limited treatment options.

## 1. Introduction

Tricuspid valve (TV) and right ventricle (RV) have emerged in recent years as major structures with important prognostic and therapeutic implications. Once called the forgotten ventricle and valve, the cardiology community came to the realization that in many patients, tricuspid regurgitation (TR) does not always regress after correction of mitral and aortic valve disease, or treatment of left ventricular (LV) dysfunction by medical therapy or revascularization with bypass surgery. Thousands of cardiologists including myself with 2 or more decades of experience and a practice spanning from the era of technical modernization to the current era, remember our mentors as saying “if you repair the left side abnormalities, TR will regress on its own” [1]. However, the experience accumulated in recent years and studies conducted in the field of valvular heart disease and cardiac surgery have demonstrated that this old vision does not hold true anymore. Tricuspid Regurgitation is now recognized as a valvular heart disease with poor prognosis if left untreated.

In this review, we begin with a case vignette that describes a patient with severe TR, its clinical manifestation, then will review pertinent studies relating the relevance of methods of evaluation and mechanism of TR before examining the result of medical, surgical and transcatheter therapy for TR. This review will also expand on recent trials with transcatheter therapy as an alternative to surgery for treatment of TR.

## 2. Case Vignette

The patient is a 57-year-old woman with past medical history of mitral stenosis for which she received a bioprosthetic mitral valve replacement at the age of 28. Subsequently, she was found with degenerative bioprosthetic mitral valve and underwent a second mitral valve replacement with a mechanical valve at the age of 42. On follow-up visits, she was found with signs of right heart failure including jugular venous distention (JVD), lower extremity edema (LEE) and ascites for which transthoracic echocardiography (TTE) demonstrated severe TR. This was associated with RV dilatation and remodeling with preserved RV function. Patient was started on medical therapy including diuretics and mineralocorticoid receptor antagonists (MRA) Spironolactone. She initially had good response to diuretics with decrease in LEE and ascites. However, she presented with recurrent signs of right heart failure. Patient was referred to the valve team for management.

## 3. Epidemiology

Valvular heart diseases (VHD) are a significant public health issue, with prevalence increasing with age and high mortality associated with the disease. Tricuspid regurgitation is a growing public health problem, as more than 4% of people >75 years old have a clinically relevant TR diagnosed by Doppler echocardiography [2]. Compared with other VHD, TR is more prevalent in women. The reason for the gender difference is not well known but it could be related to higher life expectancy and high prevalence of heart failure with preserved ejection fraction with or without atrial fibrillation (AF) in women [2, 3, 4]. Compared to mitral and aortic valve disease, the outcome of moderate or greater degree of TR is worse than that of mitral or aortic valve disease [5]. The 3-year survival is about 58% and the mortality increases with worsening of the degree of TR [6]. In an analysis of national echocardiography database, it has been shown that the mortality risk was associated with increasing grades of TR. Even mild TR was independently associated with intermediate prognosis when compared with no or trivial TR [6]. It is speculated that the excess mortality in mild TR is likely due to future risk for AF and comorbidities [6]. In patients with heart failure, with reduced and preserved ejection fraction, TR has an impact on survival [7]. Progression of significant TR over time in patients who have undergone left heart surgery without concomitant tricuspid valve surgery has an adverse impact on their outcome and has led to search of therapy for TR [4, 8].

## 4. Functional Anatomy and Physiology

**Fig. 1.**
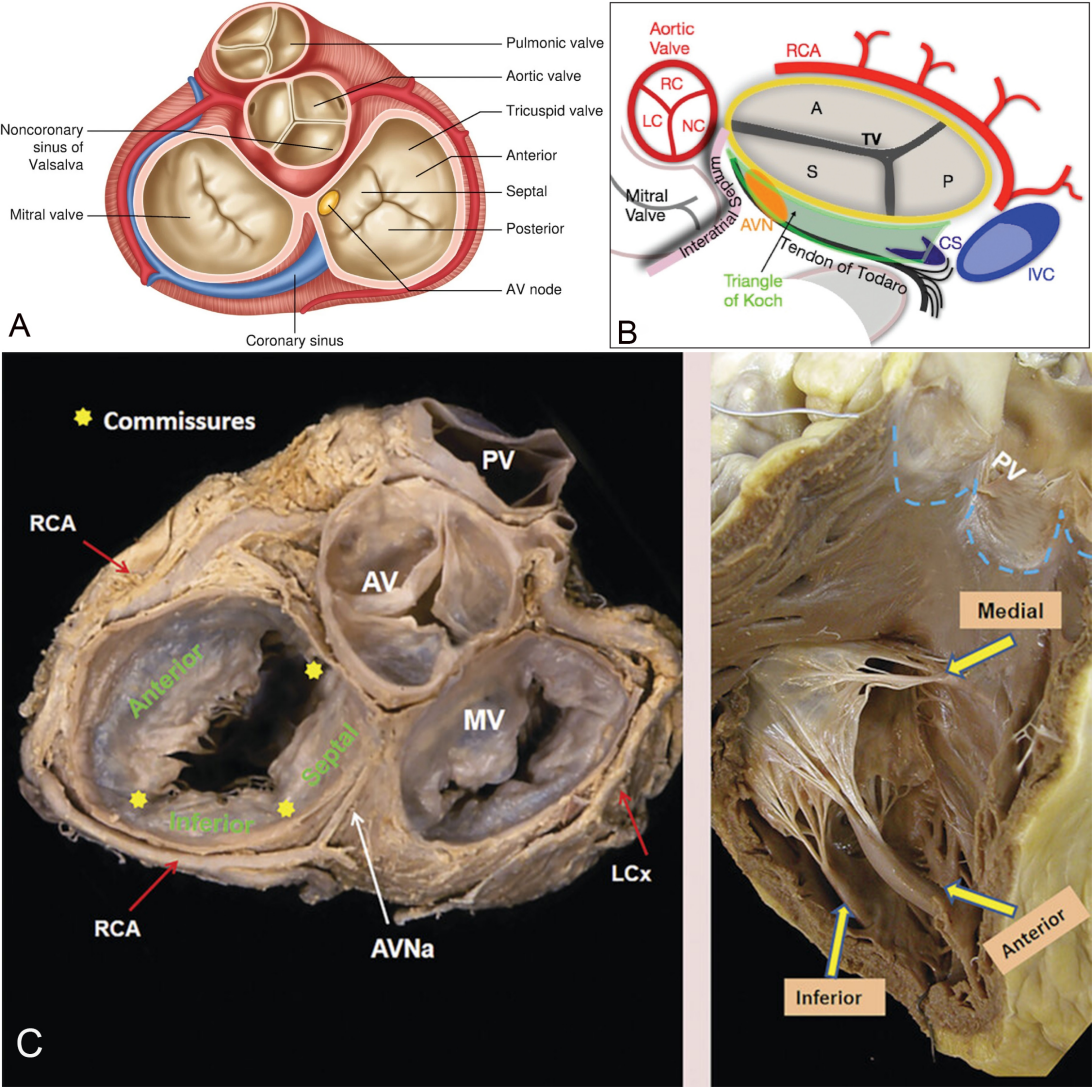
**Tricuspid valve anatomy with neighboring structures**. Schematic (A,B) and autopsy (C) demonstrate the 3 leaflets of tricuspid valve and its anatomic relationship with adjacent structures. Note the vicinity of the right coronary artery and AV node to tricuspid annulus. The proximity to AV node may cause potential complication as heart block during transcatheter intervention. The anterior papillary muscle is the principal papillary muscle and send chordae to both anterior and septal leaflets. A, anterior leaflet; P, posterior (or inferior) leaflet; S, septal leaflet; RCA, right coronary artery; CS, coronary sinus; TV, tricuspid valve; IVC, inferior vena cave; AVN, atrioventricular node; AV, aortic valve; MV, mitral valve; PV, pulmonic valve; RC, right coronary cusp; LC, left coronary cusp; NC, non coronary cusp; LCx, left circumflex artery; AVNa, atrioventricular node artery. The Fig. 1A is from the reference [9], the Fig. 1B is from the reference [10], the Fig. 1C from the link: https://radiologykey.com/17-the-tricuspid-valve-apparatus/. *Reprinted with permission from the corresponding author.*

As shown in Fig. 1 (Ref. [9, 10]), tricuspid valve is the largest of all human cardiac valves (surface area 6–8 cm^2^) and is composed of 3 unequal size leaflets with a small septal leaflet and a larger anterior and posterior leaflet. However, in about 40% of normal subjects there may be additional leaflets or doubled commissures [11]. Tricuspid valve has a saddle shape configuration with the posterior leaflet placed more inferiorly and the anterior leaflet more superiorly. For this reason, the posterior leaflet is called inferior leaflet, and the anterior leaflet is called superior leaflet by pediatric cardiologists [12]. Tricuspid valve is located anteriorly compared to other cardiac valves; a fact that needs to be considered when evaluating TR by transesophageal echocardiography (TEE).

Like mitral valve, tricuspid valve is part of tricuspid apparatus including tricuspid valve and annulus, papillary muscles, and right ventricle. There are usually 2 to 3 papillary muscles including 1 prominent anterior papillary muscle attached to the RV free wall and 2 smaller ones called posterior and septal, attached to posterior RV wall and to the septum [13, 14]. Chordae tendinea arise from papillary muscles (and sometimes from the interventricular septum) and supply the edges of tricuspid leaflets. This anatomical configuration explains why TR can occur from RV dilatation and/or chordal elongation and displacement. An important anatomical point to consider is the proximity of atrio-ventricular node and the right coronary artery to tricuspid annulus which may be at risk of injury during transcatheter or surgical valve repair. Another important point to mention is related to the structure of tricuspid annulus which contains more collagen fibers in the area where the septal leaflet is attached to tricuspid annulus compared to anterior and posterior leaflets. This difference explains why the anteroposterior and postero-septal annuli are more susceptible to dilatation under pressure and volume loading [15].

## 5. Clinical Manifestations

In the absence of pulmonary hypertension or RV failure, mild TR is generally well tolerated [16]. Patients with significant (≥ moderate) TR or pulmonary hypertension present with signs and symptoms of reduced cardiac output and right heart failure including dyspnea, reduced functional capacity, and exercise intolerance [17, 18]. When TR becomes severe, signs and symptoms of hepatic congestion and ascites develop progressively [16, 17, 18, 19]. Patients may present with ascites which may mimic liver disease [20, 21, 22]. It is not uncommon for these patients to be referred to gastroenterologists for evaluation of liver disease (personal observation). On physical exam, patients with severe TR have jugular venous distension (JVD) with prominent c-v wave and deep y wave that can be seen on inspection of jugular venous pulsation ( **Supplementary Video 1** shows a real patient with severe TR and JVD). Hepatomegaly may develop and is recognized by a soft pulsatile liver edge usually detectable by palpation. Patient may complain of right upper quadrant pain due to hepatomegaly and tension on liver capsule. Hepato-jugular reflux is another cardinal sign of severe TR. Peripheral edema is one of the prominent and earliest clinical features of RV failure. Bilateral lower extremities edema develops due to low cardiac output and backflow of blood into the systemic venous system. In early stages Antigen carbohydrate 125 and N-terminal pro-B-type natriuretic peptide may help in the detection of systemic congestion [23]. On cardiac auscultation, the murmur of mild TR may be subtle. With significant TR, the systolic murmur may be heard at the right 4th intercostal space or subxiphoid area and it increases with inspiration (Carvallo sign). However, with massive and torrential TR the murmur may become faint due to equalization of pressures in the RV and right atrium (RA). With pulmonary hypertension a loud P2 may be heard at the 2nd left sternal border. When RV failure occurs a right-side S3 gallop may be heard at subxiphoid area [23].

**Fig. 2.**
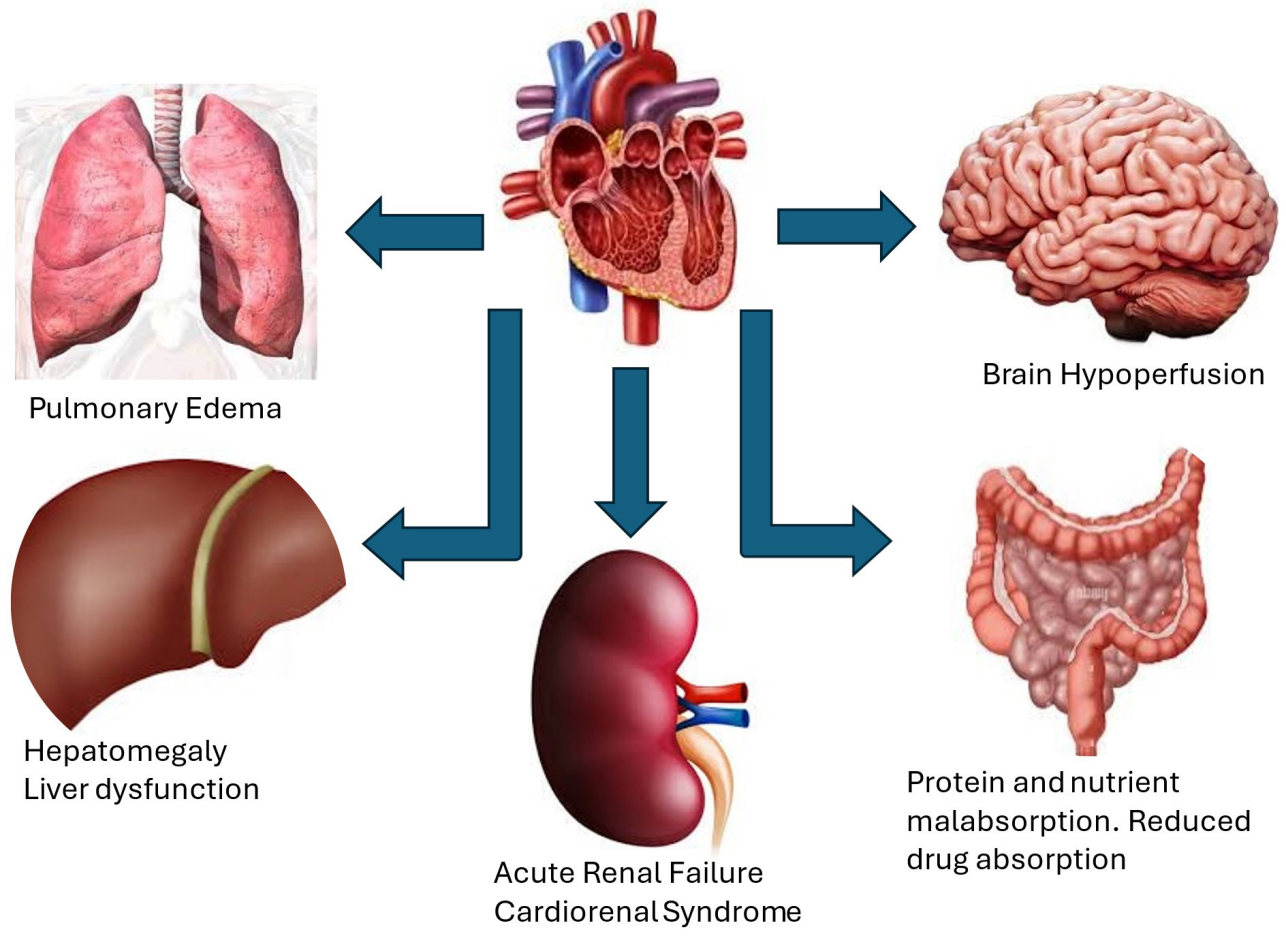
**Clinical manifestations and consequences of severe TR**. These include hepatomegaly and liver dysfunction, ascites, renal failure and cardiorenal syndrome, pulmonary congestion and edema from increased left ventricular filling pressures and reduced cardiac output due to ventricular interdependence, brain hypoperfusion, protein and nutrient malabsorption due to edema of intestinal wall resulting in cachexia.

Other clinical manifestations of severe TR are related to complications resulting from back flow of blood into the systemic venous system including hepatic congestion as mentioned above and renal failure (Fig. 2). Hepatic congestion may cause synthetic liver dysfunction with decreased protein synthesis [24]. Abnormalities in liver function include prolonged PT and elevated alkaline phosphatase and total Bilirubin and less often elevated transaminases leading to congestive hepatopathy [21, 23, 24]. Infiltration of intestinal wall with edema may cause protein losing enteropathy and eventually cachexia. The increase in renal central venous pressure and consequently the rise in renal pressure may cause worsening renal function and increase in diuretic requirement which exposes patients to diuretic resistance [25, 26]. We have previously demonstrated the role of moderate or severe TR as a risk factor in development of cardiorenal syndrome in patients with decompensated heart failure [26]. Severe TR may lead in later stages to decrease in cardiac output resulting in cerebral and peripheral hypoperfusion [27, 28]. Moreover, at later stages, ventricular interdependence leads to pulmonary congestion by shifting the interventricular septum toward the left ventricle adversely affecting LV diastolic filling and compliance resulting in increase in LV filling pressures and pulmonary edema [23, 27].

Atrial arrhythmias, especially atrial flutter and fibrillation are common in patients with moderate or greater TR and lead to left and right atrial dilatation as well as tricuspid annular dilatation which accentuates the degree of TR [29]. Restoration of sinus rhythm by electrical cardioversion or catheter ablation of atrial fibrillation may improve functional TR and promote right heart reverse remodeling [30].

## 6. Imaging Tricuspid Valve and Diagnostic Work-up

**Fig. 3.**
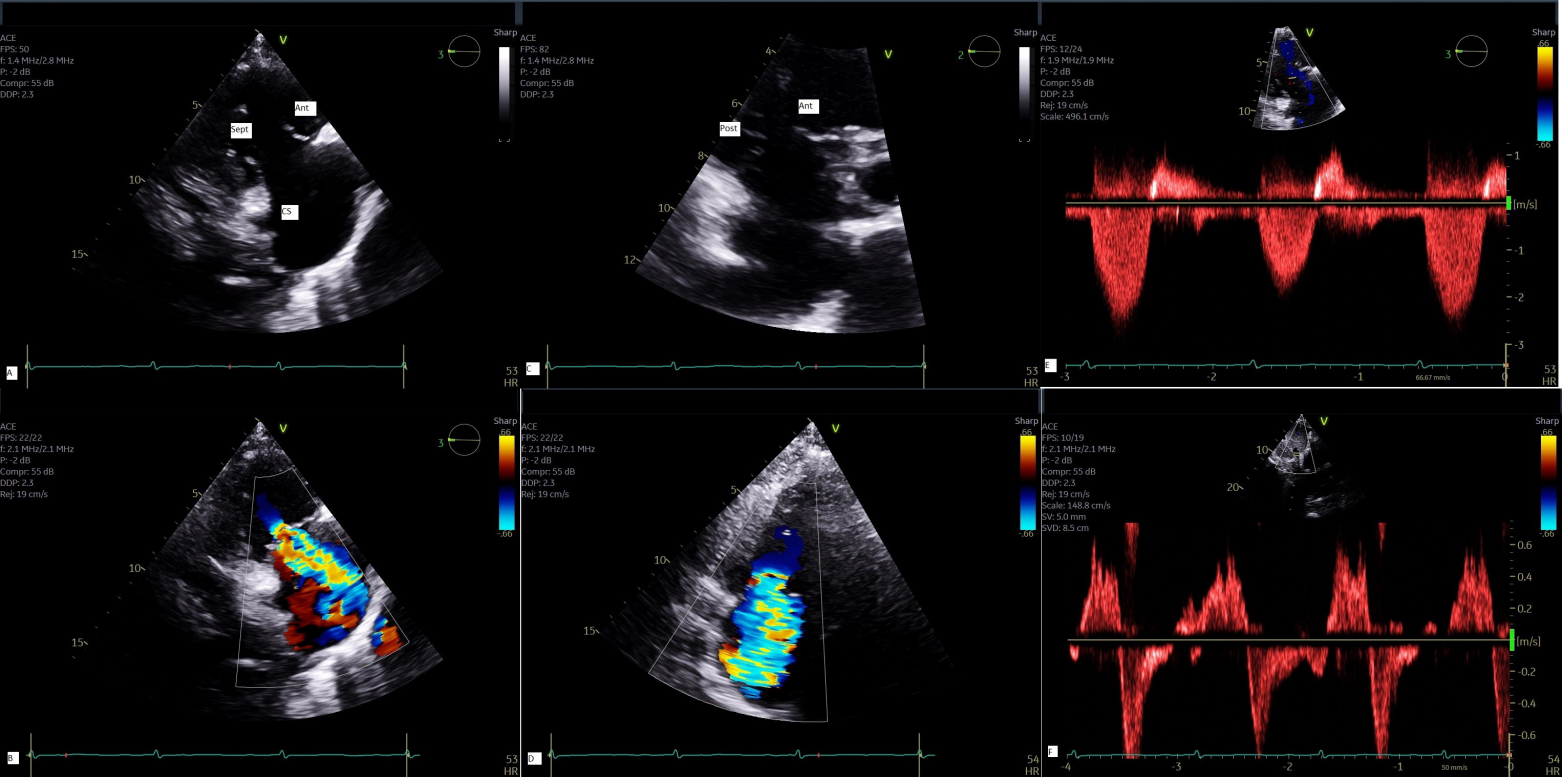
**Multi-parametric assessment of TR by TTE**. 2-D view of tricuspid valve from parasternal RV long axis (A) with color flow Doppler of TR jet (B); 2-D view of tricuspid valve from parasternal short axis (C) with color flow Doppler (D); Continuous wave Doppler demonstrates a triangular envelope (E) and systolic flow reversal in hepatic vein by pulsed Doppler (F) all characteristic of severe TR. Ant, anterior leaflet; Sep, septal leaflet; Post, posterior leaflet; CS, coronary sinus; 2D, 2-dimensional; TTE, transthoracic echocardiography.

Echocardiography is the cornerstone of imaging modality for assessment of tricuspid valve and should be performed by an experienced operator (trained sonographer or physician) at a dedicated valve center. All the echocardiography modalities including 2- and 3-dimensional echocardiography, color flow Doppler, continuous wave and pulsed wave Doppler play an important role for a comprehensive multi-parametric assessment of tricuspid valve [31]. TTE allows only visualization of 2 leaflets on a single plane in patients with adequate acoustic window due to saddle shape configuration of tricuspid valve and annulus. From parasternal long axis of the right ventricle, one can usually see the anterior and posterior leaflets. From the same view, with mild transducer rotation inferiorly one can visualize the ostium of coronary sinus with the leaflet next to it being the septal leaflet. From parasternal short axis view, at the level of aortic valve, one can see either the anterior leaflet, or anterior and posterior leaflets; with mild angulation toward the left ventricular outflow tract (LVOT), one can see the septal and part of anterior leaflets [32]. From apical 4 chamber view, the septal leaflet can be clearly visualized with the opposing leaflet being either the anterior if a portion of LVOT can be seen, or the posterior leaflet if the ostium of coronary sinus can be seen simultaneously [32] (Fig. 3 and **Supplementary Video 2**).

**Fig. 4.**
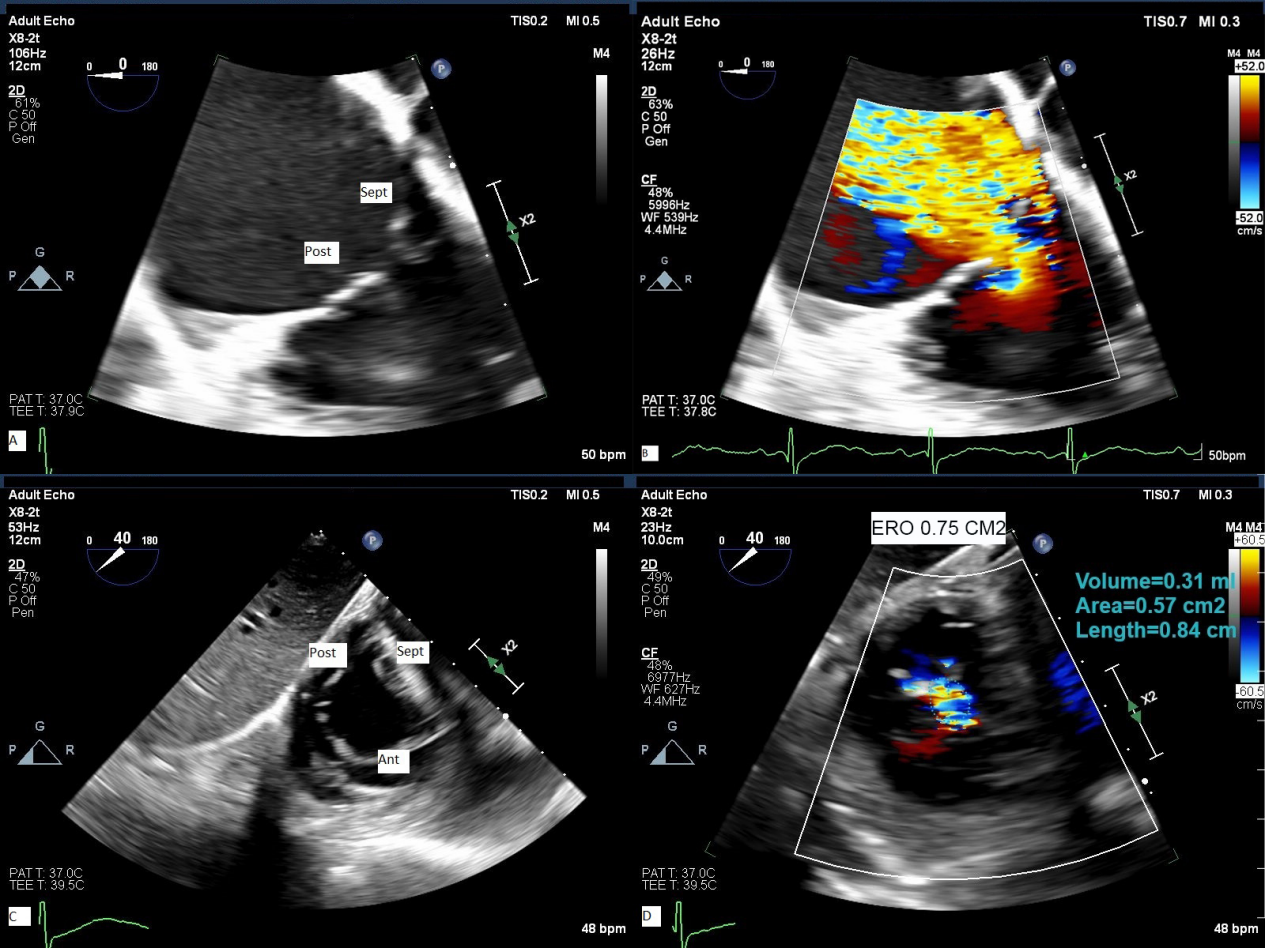
**TEE assessment of TR**. 2-D TEE from deep esophageal view (A) with color flow Doppler (B) and from trans- gastric orthogonal plane view (C) with color flow Doppler (D) demonstrate severe to massive TR. Ant, anterior leaflet; Post, posterior leaflet; Sep, septal leaflet; ERO, effective regurgitant orifice area obtained by planimetry of non-coaptation area delimited by color flow Doppler.

TEE is essential to image the tricuspid valve when transcatheter therapy is considered. Since the RV and TV are seated more inferiorly close to the diaphragm, 3 imaging planes are employed including mid esophageal, deep esophageal, and trans-gastric view with clockwise rotation of the probe. From trans-gastric view with clockwise rotation, one can visualize simultaneously the 3 leaflets by using orthogonal plane [32] (Fig. 4 and **Supplementary Videos 3–6**).

**Fig. 5.**
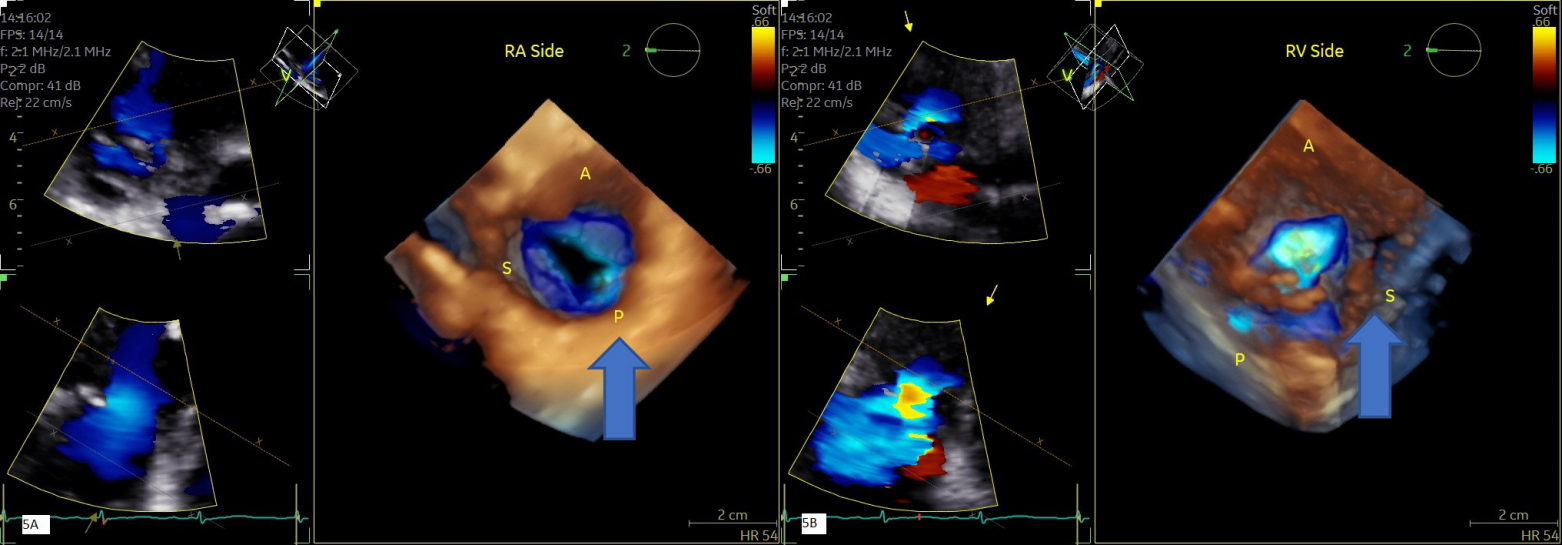
**3-D TTE assessment of TR**. image of tricuspid valve from RA side (A) and RV side with color Doppler (B) showing effective regurgitant orifice area by 3-D. See also video section. The arrow shows the non-coaptation area. 3D, 3-dimensional; RA, right atrium.

Three-dimensional Echocardiography (3-D echo) has been an important addition to imaging tricuspid valve and like for mitral valve, it obviates the need for mental reconstruction and identification of leaflets. However, the quality of 3-D echo images depends significantly on the quality of 2-D echo and since tricuspid valve is an anterior structure 3-D TTE images may sometimes have better quality than 3-D TEE images. In addition, 3-D echo systems have lower resolution than 2-D echo and higher far field attenuation which may cause 3-D imaging more challenging to acquire. At our institution, we acquire 2-D and 3-D TEE images from mid esophagus and deep esophagus position as well as trans-gastric position with clockwise rotation (Figs. 4,5). The trans-gastric view is very important for pre-procedural identification of leaflets morphology and measurement of non-coaptation area. We measure the vena contracta in orthogonal plane at mid esophageal view and effective regurgitant orifice area (EROA) from short axis trans-gastric view. The flow convergence radius is measured after reducing the Nyquist limit to ~28 cm/sec. Having part of adjacent anatomic structures such as coronary sinus and aortic valve in the acquisition field may improve the image orientation and identification of different leaflets (**Supplementary Videos 7–11**). There is a learning curve in imaging tricuspid valve and determining the degree of TR by 2-D and 3-D TEE. The American Society of Echocardiography (ASE) guidelines recommend orienting the aortic valve on the left of the frame and interatrial septum in the far field at 6 o’clock when looking at the valve from RV or RA view [33].

**Fig. 6.**
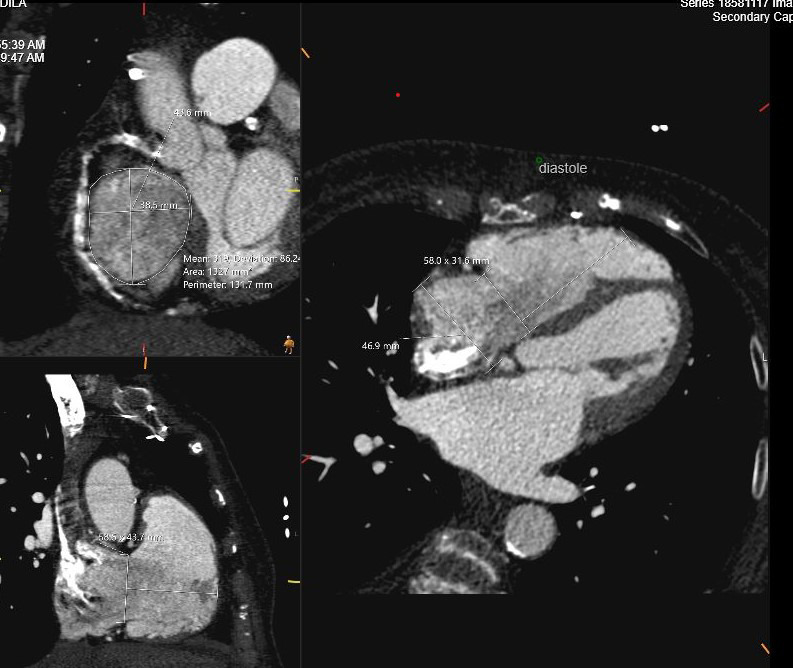
**Cardiac CT assessment of TR**. Using tricuspid valve protocol during administration of intravenous contrast in preparation for transcatheter TV replacement. Measurements include TV annulus in systole and diastole, RV height perpendicular to TV plane and RA height. TV, tricuspid valve; CT, computed tomography.

Along with TR, it is also important to evaluate RV size and function. Right ventricle is a complex structure that does not follow a geometric shape which makes RV size measurement a challenging task [34]. The guideline of the ASE recommends obtaining the RV focused apical 4-chamber view due to RV linear dimensions being dependent on probe orientation. At our institution, we evaluate RV function by at least one or a combination of parameters including tricuspid annulus plane systolic excursion (TAPSE), RV fractional area change (FAC), RV systolic velocity by tissue Doppler (RVS’), RV free wall strain (FWS) and 3-dimensional RV function [35, 36] (Table 1, **Supplementary Video 12**). Other imaging modalities with cardiac magnetic resonance (CMR) which is the gold standard modality for RV function and cardiac computer tomography (CCT) have additive role and provide complementary information on the severity of TR and RV function. Cardiac CT is performed in preparation for transcatheter TV edge-to-edge repair or TV replacement as shown in Fig. 6. A TR regurgitant volume of >45 mL and regurgitant fraction of >50% by CMR are associated with poor outcome [37]. CCT provides detailed anatomic assessment of the tricuspid annulus as well as surrounding relationship with vascular structures such as inferior and superior vena cava and right coronary artery [38, 39].

**Table 1.** **Frequently used parameters of RV systolic function with lower limit and normal values for each parameter [36]**.

Parameter	TAPSE (cm)	RV S’ (cm/sec) by tissue Doppler	FAC (%)	RV free wall strain (%)	3-D RV (%)
Normal value	≥1.7 (2.4 ± 0.3)	≥9.5 (14 ± 2.3)	≥35 (49 ± 7)	≥–20 (–29 ± 4.5)	≥45 (58 ± 8)
Advantage	Easy to obtain, reproducible, established prognostic value	Easy to obtain, reproducible, established prognostic value	Evaluate both longitudinal and radial contraction	Evaluate only longitudinal myocardial deformation, less angle and load dependent	No geometric assumptions, superior to other RV parameters, validated against MRI
Shortcoming	Evaluate longitudinal function	Angle dependent, evaluate longitudinal function only	Needs RV focus view with good image quality	Vendor dependent, post-processing, image quality, neglects RVOT contribution	Image quality and entire RV acquisition, needs special software for analysis

RV, right ventricle; TAPSE, tricuspid annulus plane systolic excursion; FAC, fractional area change; MRI, magnetic resonance imaging; RVOT, right ventricular outflow tract.

Once the diagnosis and the degree of severity of TR are established, the next step is to evaluate pulmonary artery pressures by right heart catheterization (RHC) and to differentiate pre-from post-capillary phenotypes. The degree of TR may vary significantly depending on preload and it is therefore important to optimize the volume status prior to RHC [40].

## 7. Classification of TR

**Fig. 7.**
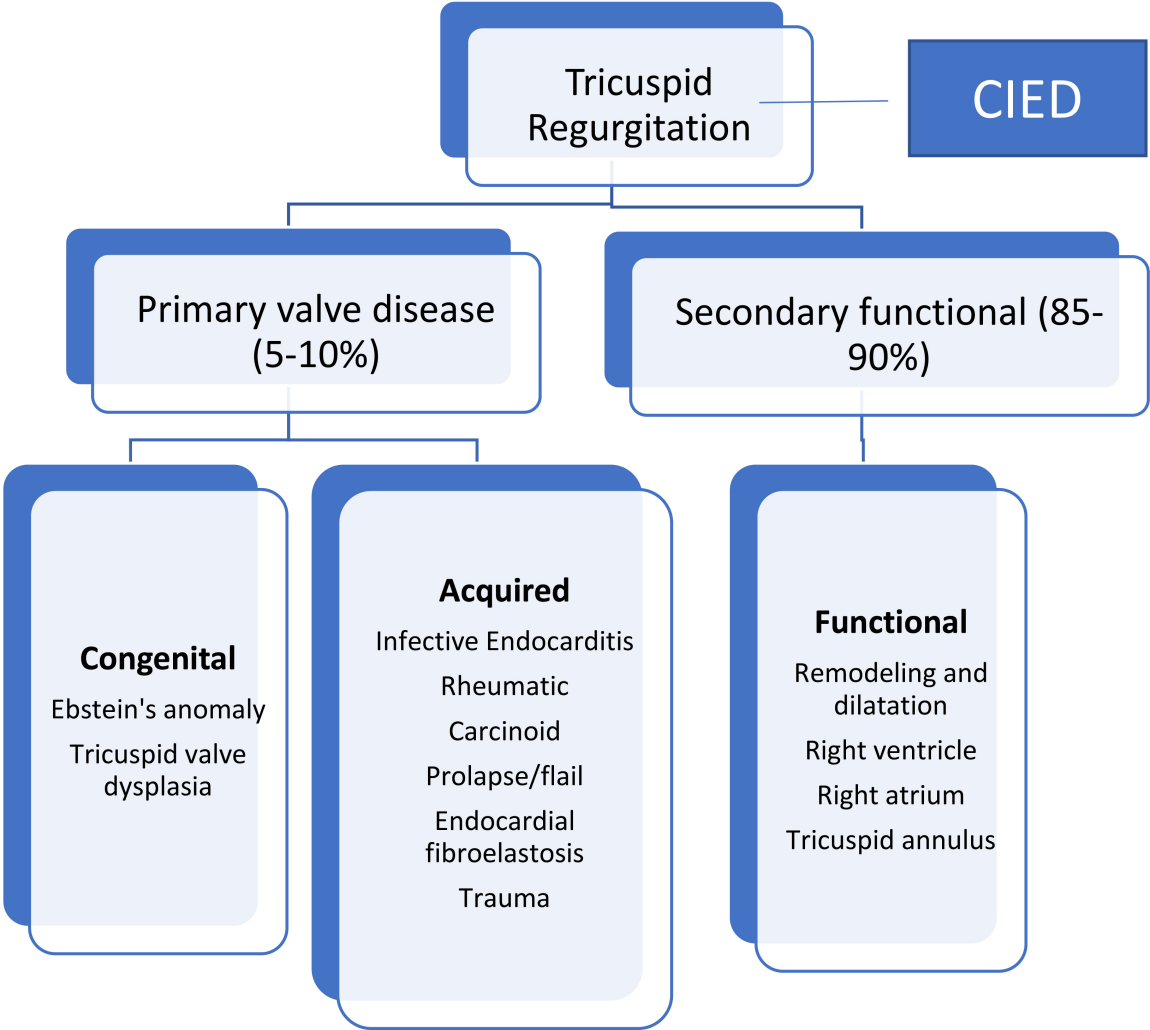
**Classification and causes of TR**. TR is divided into primary valve disease causing TR and secondary or functional TR resulting from mal-coaptation of the tricuspid valve due to RA and/or RV dilatation and remodeling. CIED, cardiac implantable electronic device.

TR is classified into primary tricuspid valve disease leading to regurgitation and secondary or functional TR due to alteration in geometry of tricuspid valve apparatus [38, 39]. Primary TR accounts for 5–10% of cases and is caused by primary structural alterations of tricuspid valve apparatus that can be either acquired or congenital. Secondary TR (85–90% of cases) is the most common phenotype encountered in adult patients [23] and is subdivided into atrial and ventricular functional TR because of their different prognostic implications [41, 42] (Fig. 7). Although this classification is important when considering therapy for functional TR, in many instances valvular regurgitation may lead to progressive RV and RA dilatation and geometric changes and results in a mixed phenotype with both RV and RA dilatation. Coexistence of TR with severe mitral regurgitation in 30 to 50% of patients and with severe aortic stenosis in 25% of patients is well established [43]. In addition, coexistence of TR with mitral and aortic regurgitation may adversely affect short- and long-term outcomes [43]. A third category of TR is associated with cardiac implantable electronic devices (CIED) which has been growing in incidence due to increasing indication and use of CEID (see below).

## 8. Primary Tricuspid Valve Disease Causing TR

Ebstein’s anomaly which may have a genetic basis (mutation in the *MYH7* gene) represents the most frequent congenital abnormality of tricuspid valve in which tricuspid leaflets are tethered to the RV myocardium causing leaflets non coaptation and TR [12, 44]. The mechanism of TR in Ebstein’s anomaly is a defect in the process of delamination of leaflets, so that leaflets remain tethered to RV myocardium to a variable degree [12]. In addition, there is anterior displacement and downward rotation of tricuspid leaflets causing a non-coaptation orifice. The degree of TR and atrialization of RV are variable among patients with Ebstein’s anomaly and determine the severity and age of clinical manifestations [45].

The incidence of tricuspid valve endocarditis correlates with that of intravenous drug abuse which is unfortunately on the rise. Vegetations can cause valvular lesions, perforation, valvular abscess and may extend to chordae causing chordal rupture, all of which may cause impairment in valvular coaptation leading to various degree of TR [46].

Tricuspid valve prolapse due to myxomatous degeneration is rare and usually associated with mitral valve prolapse. Trauma to tricuspid valve can occur after blunt chest trauma such as motor vehicle accident and trauma from kick to the chest in martial sport. Recurrent RV myocardial biopsy in patients after heart transplant can cause flail tricuspid valve leading to severe TR [47]. Rheumatic tricuspid valve disease is usually associated with rheumatic mitral valve disease and may cause either stenosis or regurgitation or both [48]. Carcinoid heart disease is a rare condition that is part of carcinoid syndrome and associated with carcinoid tumors and metastasis to the liver [49]. Carcinoid tumors secrete toxic substances such as serotonin which causes fibrosis and retraction of tricuspid leaflets and lead to characteristic fixed immobile leaflets and severe TR seen by echocardiography. TR in carcinoid valve disease is often associated with acquired pulmonic stenosis as well [50].

The entity of TR associated with CIED has been refined recently and involves impingement or perforation of the valve leaflets by an implantable intracardiac device leading to TR [51]. The degree of TR in patients with implantable intracardiac devices varies from mild to severe and sometimes more. The diagnosis can be made with the use of 3-D echo by demonstrating the intracardiac wire causing impairment in tricuspid’s leaflet opening and closing properly [52].

## 9. Secondary or Functional TR

**Fig. 8.**
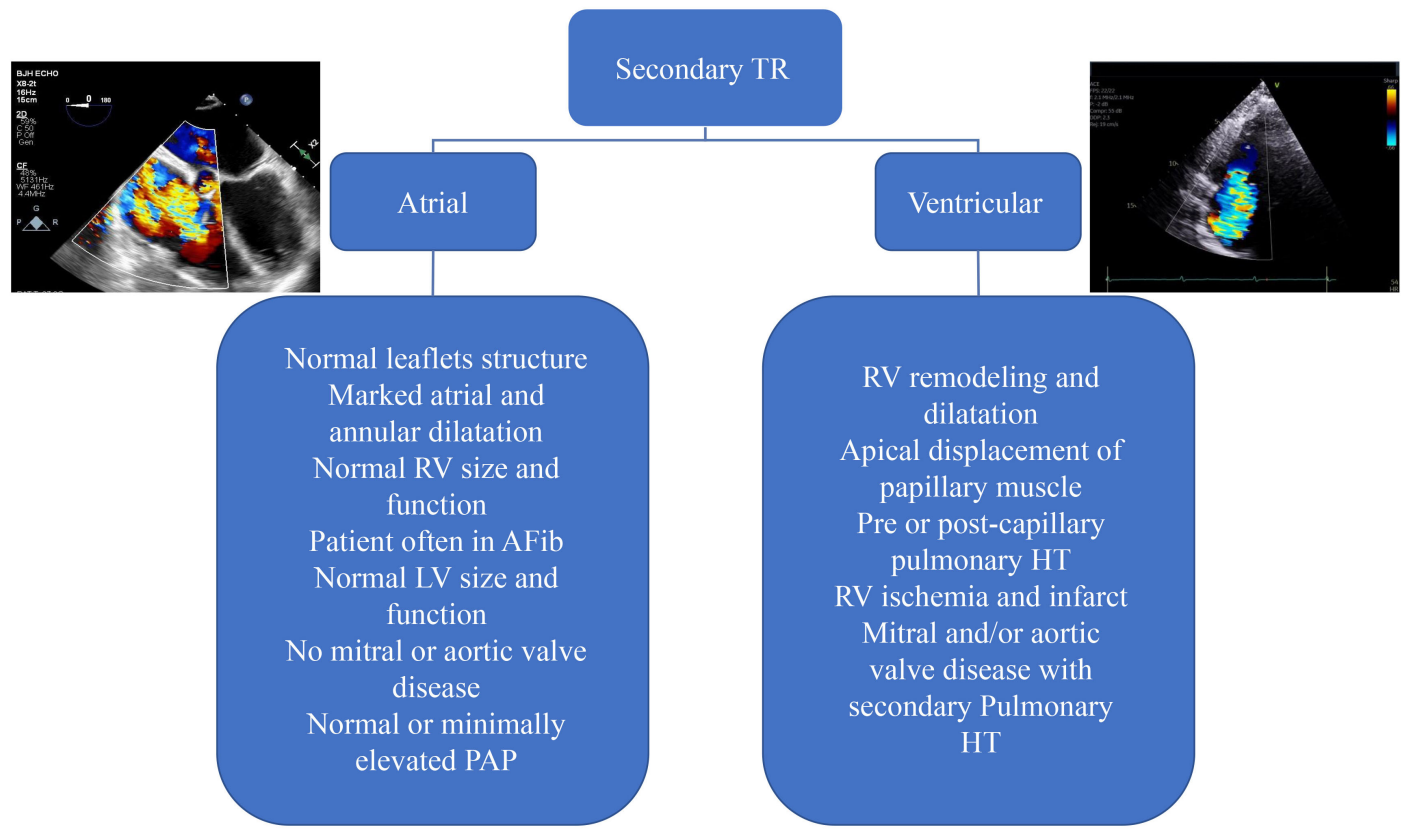
**Classification and causes of functional tricuspid regurgitation into atrial and ventricular causes**. In atrial TR, the primary mechanism is the dilatation of the RA and tricuspid annulus. Whereas, in ventricular TR, the primary cause of TR is RV dilatation and remodeling which could be a consequence of long-standing left-sided myocardial or valvular disease. In advanced stages a combination of atrial and ventricular TR is often encountered. LV, left ventricular; PAP, pulmonary arterial pressure; pulmonary HT, pulmonary hypertension.

Secondary TR is the most common cause of TR encountered in clinical practice and is subdivided into atrial and ventricular TR [53]. However, once TR becomes significant enough to cause symptoms, every component of tricuspid apparatus may be involved in the mechanism of TR including dilatation and remodeling of the RA and RV, displacement of papillary muscles and tricuspid annulus dilatation [54] (Fig. 8). The rationale behind dividing secondary TR to atrial and ventricular mechanism is that their prognosis and therapeutic implications differ [55, 56]. Patients with ventricular TR phenotype and either severe pulmonary hypertension or left heart disease have higher mortality than patients with atrial TR [57]. Patients with ventricular secondary TR have 2.7- fold higher risk of experiencing the combined endpoint of death and hospitalization for heart failure than patients with atrial TR phenotype [57]. However, once RV function deteriorates, differences in the outcomes of patients with atrial and ventricular severe TR becomes less pronounced [58].

## 10. New Grading System for Assessment of TR Severity 

Transthoracic echocardiography remains the first imaging modality to evaluate the degree of TR with multiparametric assessment including color flow Doppler as previously mentioned. It is important to bear in mind that pressures in the right heart are lower and therefore the evaluation of TR by color Doppler only may underestimate the severity of TR. For this reason, a multi-parametric approach including continuous wave Doppler of TR jet and pulsed Doppler of hepatic vein, inferior vena cave(IVC) size and respiratory variation has been proposed to better evaluate the severity of TR and to address the degree of TR beyond severe. Color flow Doppler remains the primary modality for evaluation of the degree of TR. Understanding principles behind color flow jet area is important since it is governed primarily by jet momentum and machine settings. Jet momentum itself depends on flow rate and blood flow velocity. In general, a jet area of >10 cm^2^ in the RA and a vena contracta >0.7 cm suggest severe TR (Table 2, Ref. [59]).

**Table 2.** **New grading system for TR with color flow Doppler assessment**.

Variable	Mild	Moderate	Severe	Massive	Torrential
Vena Contracta width (mm)	<3	3–6.9	7–13	14–20	>20
EROA by PISA (mm^2^)	<20	20–39	40–59	60–79	>80
EROA by 3D (mm^2^)			75–94	95–114	>115
Regurgitant Volume (mL/beat)	<15	15–29	40–59	60–74	>75

EROA, effective regurgitant orifice area; PISA, proximal isovelocity surface area (Ref. [59]).

The new grading system was developed because many patients with secondary TR were presenting in a stage of advanced heart failure having massive or torrential TR [60, 61]. It has also been shown that quantitative assessment of TR, particularly EROA, is the most powerful predictor of outcome and superior to standard qualitative assessment. Peri *et al*. [62] showed that the best discriminator value for severe TR was an EROA >0.35 cm^2^ using Cox hazard analysis and EROA >0.30 cm^2^ using receiver operating characteristic (ROC) curves for 1 year mortality similar but slightly lower than guidelines proposed EROA of 0.4 cm^2^. This grading system showed to be useful in terms of reporting the efficacy of transcatheter reduction of the degree of TR and clinical benefits of such devices.

## 11. Current Therapy for TR

### 11.1 Medical Therapy 

The first step in the treatment of secondary TR is medical therapy with diuretics to optimize the volume status. Loop diuretics and mineralocorticoid receptor antagonists (MRA) are used to treat volume overload in patients with significant TR regardless of left ventricular ejection fraction (LVEF) [63]. Although an adequate initial response with decrease in volume overload and the degree of TR are expected in most patients, diuretic resistance and worsening kidney function may develop and is associated with poor prognosis [26, 64]. A combination of loop and thiazide diuretics has the potential to increase natriuresis and help with volume overload. Mineralocorticoid receptor antagonists have an impact in reducing RV afterload [65]. Moreover, in a small randomized controlled trial including patients with heart failure and reduced ejection fraction, sodium-glucose cotransporter 2 inhibitors (SGLT-2) in addition to other guideline directed medical therapy for heart failure were found to be more effective in improving RV function as compared to other heart failure drugs alone [66]. Once pulmonary hypertension is detected by echocardiography, RHC is recommended to measure the pulmonary artery pressure and to differentiate pre from post-capillary pulmonary hypertension in patients with severe TR regardless of left heart disease before considering trans-catheter or surgical intervention on tricuspid valve [67]. Pulmonary artery pressure may be underestimated by echocardiography when the degree of TR is severe or more than severe in which case RHC should be considered [67]. Some studies have reported improvement in TR severity and RV remodeling and biomarkers after guideline directed medical therapy of left heart disease. This is especially true in patients with mitral regurgitation associated with left ventricular systolic and diastolic dysfunction and after transcatheter edge to edge repair of mitral valve, although no mortality benefit with medical therapy has been demonstrated [67, 68]. Current guidelines clearly state that medical therapy should not delay TR intervention when indicated [69].

Atrial fibrillation is a common arrhythmia in patients with severe secondary atrial TR and restoration of sinus rhythm either by electrical cardioversion or catheter ablation results in improvement in atrial volumes and reduction in TR and should be tried before considering surgical and transcatheter therapy [70, 71]. Restoration of sinus rhythm by maze procedure also can halt the progression of TR after mitral valve surgery [72].

### 11.2 Surgical and Trans-Catheter Therapy

**Fig. 9.**
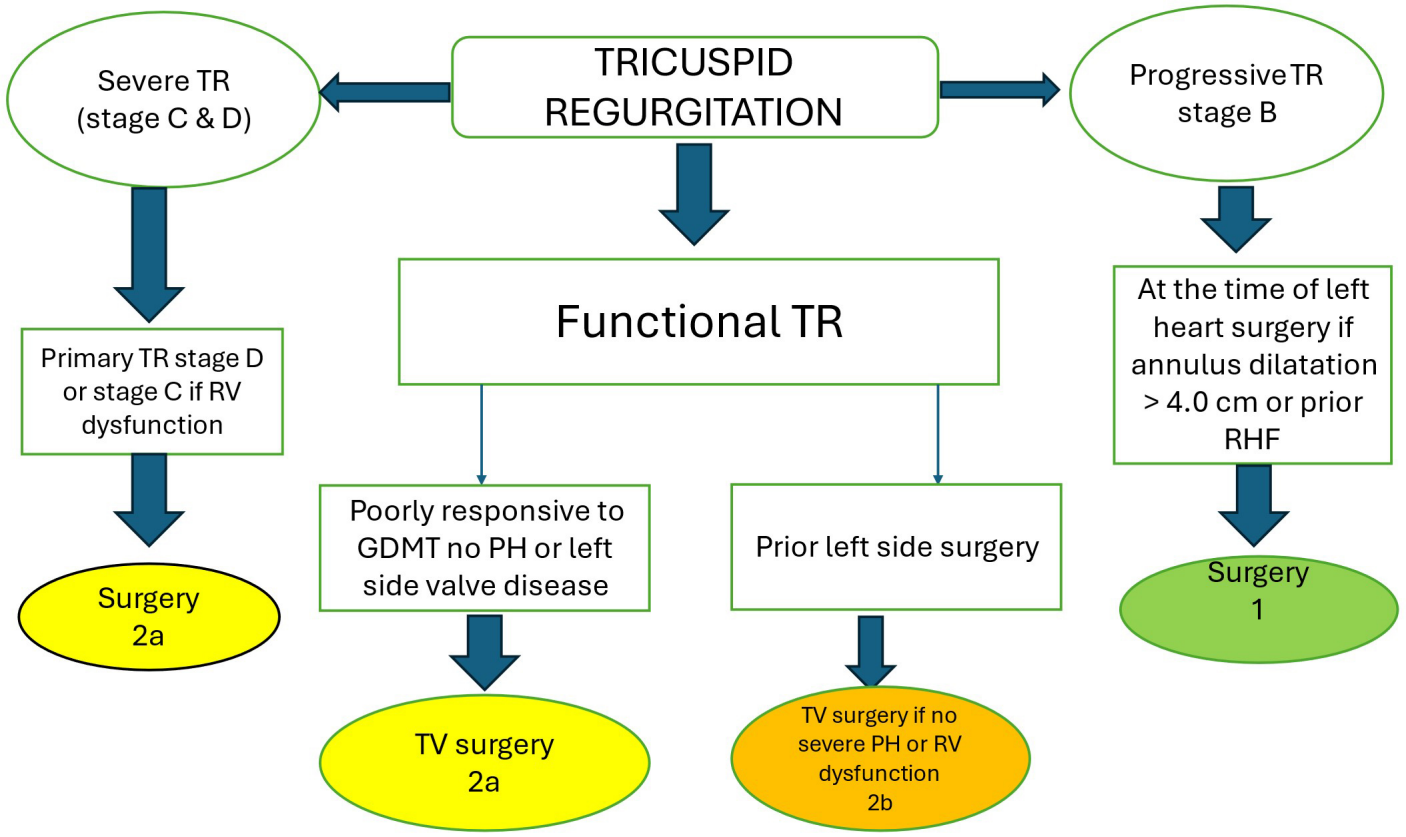
**Simplified algorithm for surgical management of patients with TR (adapted from Ref [74])**. 1 = strong class of recommendation; 2a = moderate class of recommendation (reasonable); 2b = moderate class of recommendation (may be reasonable). RHF, right heart failure; GDMT, guideline directed medical therapy; PH, pulmonary hypertension.

Experience acquired over the last 2 decades taught us that surgical treatment of left heart disease does not necessarily improve associated significant TR [73]. Indications for treatment of TR are based on the severity of regurgitation (grading), as well as on the presence of signs and symptoms of right-sided heart failure and on the extent of tricuspid annular dilation, leaflet tethering, and pulmonary hypertension (staging of disease). In terms of timing and indications for intervention on TR, current guidelines of the American College of Cardiology and American Herat Association (ACC/AHA) give a class I recommendation for surgical treatment of severe TR (stages C and D) at the time of left-sided valve surgery [73, 74]. For patients with symptomatic severe primary TR (stage D) and those with isolated severe secondary TR who failed medical therapy (stages C and D), the guidelines give a class IIa recommendation, in the absence of pulmonary hypertension. Class IIa recommendation applies as well for patients with progressive TR (stage B) undergoing left-sided valve surgery if tricuspid annulus end-diastolic diameter is >4.0 cm or in patients with prior signs and symptoms of right sided heart failure (Fig. 9) [74]. Due to high risk of mortality (10–25%) associated with reoperation in patients with previous left heart surgery, the guidelines give a class IIb recommendation (isolated tricuspid valve surgery can be considered) for surgical treatment of severe symptomatic primary or secondary TR in the absence of pulmonary hypertension or RV dysfunction. Because of dynamic nature of functional secondary TR depending on preload and pulmonary pressures, a prior history of right-sided HF indicates the propensity to develop recurrent severe TR and should be considered as an indication for concomitant tricuspid valve repair [74].

The choice between surgical tricuspid annuloplasty repair and valve replacement depends on the degree of tricuspid leaflets tethering and RV dilatation and dysfunction. At our institution tricuspid valve annuloplasty repair is preferred to valve replacement if the degree of leaflets tethering is not significant. Otherwise, the outcome is more favorable with valve replacement in patients with severe leaflets tethering and marked RV dilatation with tricuspid annulus larger than 44 mm [75].

### 11.3 Transcatheter Therapy

Given the high mortality rate in patients with severe symptomatic secondary TR who have previously had left heart surgery [76, 77], transcatheter therapy has emerged as a viable and promising alternative to surgery in these patients. Table 3 (Ref. [78, 79, 80, 81, 82, 84, 86, 89, 91]) summarizes pertinent studies on transcatheter interventions for TR. Transcatheter therapy, particularly edge-to-edge repair devices (Mitral Clip and TriClip), aiming to approximate the leaflets have demonstrated promising results for safety, reduction in TR severity, and improving the quality of life although many challenges still exist due to complex anatomy of TV apparatus [87].

**Table 3.** **Overview of pertinent studies on transcatheter intervention for TR (see text for details)**.

Study and Author	Device used	Number of patients	Follow-up duration	TR reduction	Functional improvement
Nickenig G. *et al*. (Ref. [78])	MitraClip	64	30 days	91% of patients; 1 grade TR reduction	6-min. walk and quality of life
TRILUMINATE	TriClip system	85	12 months	71% of patients with	NYHA class I or II in 83% of patient, improvement in 6-min walk and KCCQ point
Lurz P. *et al*. (Ref. [79])				reduction of TR to moderate or less	
Fam N.P. *et al*. (Ref. [80])	PASCAL system	28	30 days	85% of patients with TR grade <2+	Improve in 6 min walk distance
Kodali S. *et al*. (Ref. [81])	PASCAL system	34	30 days	TR reduction to grade 2 or less	Quality of life, exercise capacity, functional status
Nickenig G *et al* (Ref. [82])	Cardioband	30	Up to 2 years	72% of patient with < moderate TR	Improved NYHS class; 6-min walk improved by 73 m and KCCQ by 14 points
Hahn RT *et al*. (Ref. [89])	Gate TTVI system	30	172 ± 82	76% of patients with mild or less TR	62% in NYHA class I or II
Webb JG *et al*. (Ref. [84])	Evoque TTVI system	27	1 year	96% TR ≤2, 87% TR ≤1	70% of patients in NYHA class I or II
Kodali S *et al*. (Ref. [91])/TRISCEND Trial	Evoque TTVI system	176	1 year	98% with mild or no residual TR	78.8% in NYHA class I or II; 6-min walk +48 m, KCCQ +19 points
Estévez-Loureiro R. *et al*. (Ref. [86]) Tricus Euro	TricValve system	35	30 days success 94%	To reduce the systemic effects of severe TR	At 6 months 79% in NYHA class I or II and improved KCCQ

KCCQ, Kansas City Cardiomyopathy Questionnaire; NYHA, New York Heart Association.

**Fig. 10.**
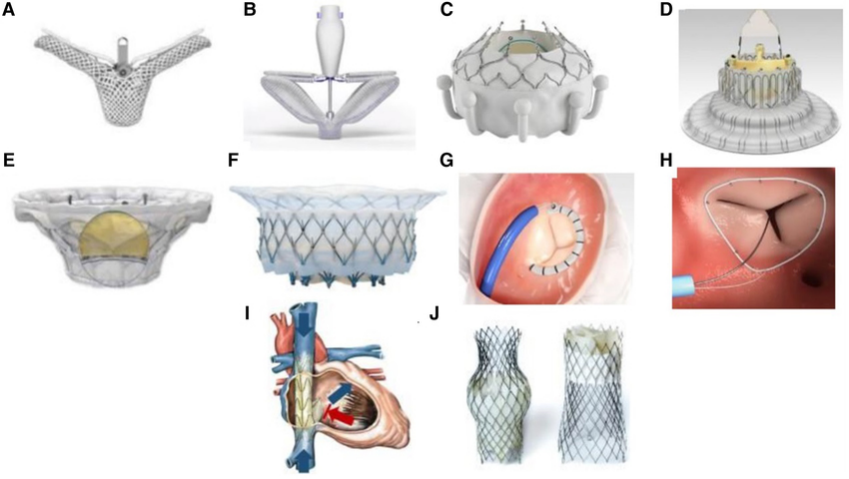
**Transcatheter tricuspid valve devices**. (A) TriClip (Abbott Vascular, Santa Clara, California, USA). (B) PASCAL system (Edwards Lifesciences, Irvine, California, USA). (C) EVOQUE system (Edwards Lifesciences, Irvine, California, USA). (D) LuX-Valve (Jenscare Biotechnology Co., Ningbo, China). (E) Cardiovalve (Boston Medical, Shrewsbury, MA, USA). (F) Intrepid valve (Medtronic Plc, Minneapolis, MN, USA). (G) Cardioband tricuspid valve reconstruction system (Edwards Lifesciences, Irvine, California, USA). (H) Tri-Ring annuloplasty system (Cardiac implants, California, USA. (I) TRICENTO system (Medira AG, Balingen, Germany). (J) TricValve (NVT, Muri, Switzerland). The Fig. 10 is from the reference [88]. *Reprinted with permission from the corresponding author.*

Other coaptation devices and prosthetic valves have been developed commercially to address the mal-coaptation of leaflets and annular dilatation (Fig. 10, Ref. [88]). Early feasibility study of a transcatheter tricuspid valve edge-to-edge repair enrolled 64 patients in New York Heart Association (NYHA) class >II with moderate or greater TR and used MitraClip system for compassionate use to reduce TR. The trial confirmed the safety of the device and improvement in quality of life [78]. Subsequently, it was shown that a reduction in TR severity of 1 grade was indeed associated with short term improvement of symptoms and quality of life. The result of TRILUMINATE trial, a prospective multicenter trial on 85 patients with moderate or greater TR and in NYHA class II or higher, was published in 2021 and showed the safety of TriClip device used in this trial and reducing TR by at least one grade in 71% of patients at 1 year follow-up [79]. The PASCAL transcatheter valve repair system used a device with a central spacer to occupy the regurgitant orifice area to increase the procedural safety and minimize stress on TV leaflets. The system was used in a multi-center early compassionate experience on 28 patients with severe TR and demonstrated safety and high procedural success of the device [80]. Subsequently, PASCAL system was used in 34 patients with symptomatic severe or greater TR and showed substantial TR reduction to less than grade 2 and a significant improvement in functional status, exercise capacity and quality of life at 30 days follow-up [81]. In addition, the 6-month outcomes of the transcatheter edge-to-edge repair using MitraClip system has demonstrated that the reduction of TR was associated with significant RV reverse re-modeling and improvement in RV function [87]. Tricuspid annuloplasty systems are based on the same principal as surgical annuloplasty with the goal of reducing tricuspid annulus diameter. The currently approved system uses tricuspid annuloplasty device Cardioband® which is a catheter-delivered annular reduction system that mimics the surgical annuloplasty approach. The system has demonstrated improvement in NYHA functional class at 2 years follow-up in 75–80% of patients in multi-center TRI-REPAIR study [82]. The most common procedure associated complications were bleeding and complications due to proximity to the right coronary artery [82]. Tricuspid valve replacement systems use a bioprosthetic valve delivered via a catheter system. In this technique we mention the GATE valve system and the EVOQUE. The GATE system was the first transcatheter valve used as compassionate indication in few patients with severe TR not candidate for surgical valve replacement [83]. Subsequently, The Gate system was used in other studies with larger number of patients and showed a high procedural success rate of >90% and reduction in the grade of TR of 1 or 2 grades [89]. When using these systems, there is less concern about valve anatomy. The EVOQUE valve system was used first in a compassionate trial in 27 patients at high surgical risk. At one year, 70% of patients were in NYHA class I/II [84]. In TRISCEND trial, 56 patients received Evoque valve. At 30 days follow-up in this trial, 98% of patients had mild or no TR and 78.8% were in NYHA class I and II. The 6- minute walk distance has improved by 48 meter and Kansas City Cardiomyopa-thy Questionnaire (KCCQ) improved by 19.0 points. The composite major adverse event rate was 26.8% including 1 death, 15 major bleeding and 2 device embolization [85]. The evidence from the clinical trials to date utilizing transcatheter valve replacement suggests that these techniques are safe with high success rate and associated with reduction of at least 1 grade of TR severity and more importantly they can improve patient’s symptoms, functional capacity and NYHA functional class at one year follow-up [90, 91]. Another device therapy used in reducing the severity of TR and indirectly treating the systemic effects of severe TR is called heterotopic caval valve implantation using dedicated specifically designed self-expandable (TricValve and Tricento) or balloon expandable valves (Sapien). These valves are deployed in the superior vena cava (SVC) and IVC or just in the IVC (Tricento) where they can reduce caval flow reversal due to severe TR. In TRICUS EURO study, TricValve was used in 35 patients with improvement in both NYHA functional class and KCCQ without peri-procedural deaths [86].

Overall, transcatheter tricuspid valve repair and replacement appear to be safe and effective in improving symptoms and quality of life in a large percentage of patients during follow-up of 12 months. The real-world outcome for tricuspid edge-to-edge repair from the BRIGHT trial was presented at PCR London Valves in 2022. At one year, 86% of patients had moderate or less TR. The improvement in NYHA functional class and KCCQ were maintained during the same period and were associated with 44% reduction in hospitalizations. However, there was also 11% mortality rate [92].

The question which remains is what the outcome of transcatheter intervention compared to surgery would be in randomized clinical trials with longer duration of follow-up?

## 12. Conclusion

Tricuspid regurgitation is now recognized as a major valvular heart disease with poor prognosis if left untreated. Many patients remain asymptomatic and present with severe or greater degree of TR and many of them have already had left-sided valve surgery or coronary artery bypass grafting. It is in this context that transcatheter therapy has become a major viable alternative to re-do surgery in the management of patients with severe or greater TR. Because of diversity of commercially available devices and techniques, it is important to adopt an approach that is personalized to every patient’s anatomy and mechanism of TR for the expected success of the intervention to be maximal. Patient selection for transcatheter or surgical intervention is very important and should be considered by a multi-disciplinary team including interventionalist, cardiac surgeon, and imaging specialist with expertise in structural echocardiography. It has been shown that major determinants of success in transcatheter edge-to-edge repair (TEER) devices are complexity of leaflets structure, leaflet coaptation gap, the location of TR jet and the degree of annular dilatation and leaflets tethering [93].

The patient in our case vignette received transcatheter Evoque valve replacement with only trace residual TR (**Supplementary Videos 13,14**). The patient has not been admitted for recurrent heart failure exacerbation since the valve replacement and was in NYHA class II at her last follow-up 6 month after TV replacement.

## 13. Future Direction

It is expected that transcatheter interventions for severe TR will expand in the coming years parallel to increasing demand as many patients may be at high risk for open-heart surgery. As biotechnology industry generates more efficient devices, there will be an increasing need of high level of training in structural interventionalists and echocardiographers for these procedures to be performed in a safe and efficient manner.

## Supplementary Material

Supplementary material associated with this article can be found, in the online version, at https://doi.org/10.31083/RCM28173.
